# Protective effect of vitamin E on oxidative stress and sperm apoptosis in diabetic Mice

**DOI:** 10.18502/ijrm.v17i2.3990

**Published:** 2019-03-20

**Authors:** Khadijeh Mirzaei Khorramabadi, Ali Reza Talebi, Abolghasem Abbasi Sarcheshmeh, Aghdas Mirjalili

**Affiliations:** ^1^International Campus, Shahid Sadoughi University of Medical Sciences, Yazd, Iran.; ^2^Research and Clinical Center for Infertility, Yazd Reproductive Sciences Institute, Shahid Sadoughi University of Medical Sciences, Yazd, Iran.; ^3^Department of Biology and Anatomical Sciences, Shahid Sadoughi University of Medical Sciences, Yazd, Iran.

**Keywords:** *Case-control study*, *Vitamin E*, * Diabetes treatment*, *Diabetic Syrian mice*, * Male reproductive dysfunction*

## Abstract

**Background:**

Generation of free radicals and oxidative stress are a major contributor to diabetes. These factors lead to the development of diabetic testicles disorders.

**Objective:**

In this study, the protective effect of vitamin E on functional disorders associated with diabetes induced oxidative stress in male reproductive systems has been investigated.

**Materials and Methods:**

Thirty-three adult male Mice were divided into control, diabetic, and untreated diabetic groups. Streptozotocin was used to induce diabetes. In the treated group, vitamin E was given to the Mice intraperitoneally for 30 days. Then, animals were anesthetized and sacrificed. Animal testicles were isolated and homogenized in phosphate buffer and used for measuring sperm count, motility and survival of sperm, MDA concentration and antioxidant capacity (TAC). Apoptosis was also performed with the TUNEL test.

**Results:**

The results of reduction (12.03±98.11) TAC, MDA concentration (–28.5±2.58), sperm motility (unstable sperma= 86.4±7.48), sperm count (171.51), Sperm morphology (natural morphology= 49.69±31.93) and abnormal morphology (9.77±49.7) with increased oxidative damage. These changes were statistically significant in comparison with the control group for all variables other than MDA (p= 0.05). Treatment of vitamin E diabetic Mice improved the ability of antioxidants to prevent oxidative damage in the testicles, restore the sperm movement, and increase the number of normal sperm as well as TAC. The level of apoptosis in the treated group has decreased compared to the untreated group.

**Conclusion:**

Vitamin E protects the reproductive system against diabetes mellitus. Therefore, it was concluded that vitamin E may be a suitable agent for protecting the sperm and testicular parameters against undesirable effects of diabetes.

## 1. Introduction

Diabetes mellitus (DM) is a metabolic disease that is closely associated with male reproductive dysfunction (1). The production of Free radicals and oxidative stress is an important pathogenic factor in diabetes (2). It is shown that there is a direct relationship between DM, fertility and pregnancy losses. On the other hand, the age of the patient is very important. In fact, the percentage of patients with type 2 DM is four times more than type 1 DM in japan. In the America, more than one-third of the new DM diagnostics are in young ages. Although, it is demonstrated that the pathological and biochemical changes due to the DM are responsible of reduction in male fertility (3). These factors lead to the development of diabetic testicular dysfunction. The imbalance between reactive oxygen species (ROS) production and body antioxidant defense cause oxidative stress (OS) which is very important in sperm fertility potential (4). This imbalance promotes the process of apoptosis because of the crosstalk between oxidative stress and apoptosis (5). The most important ROS are hydrogen peroxide and proximal. Hydroxide and hypochlorous acid are other non-oxygen radicals produced by diabetes. Reactive nitrogen species including oxide nitrite and nitrite proxy also have important biological activity. ROS and RNS are continuously produced in the physiological cycle. Uncontrolled production of intracellular ROS can damage macromolecules (DNA, lipids, and proteins) (6). In order to reduce these destructive functions, there are many intra- and extra-cellular antioxidant defense mechanisms to neutralize the effects of free radicals (7, 8). Vitamin E, carotenoids, lipoic acid, selenium, etc., also help to remove the produced free radicals (9). The most important free radicals in human semen are superoxide anion radicals, hydrogen peroxide, and hydroxyl radicals (10). These free radicals are typically produced during oxygen metabolism. Under physiological conditions, low level of ROS is essential for normal function of sperm (11). However, the production of excessive amounts of ROS can cause serious damage in sperm. Recent studies have reported that there are high levels of ROS in 25–40% of infertile men that leads to damage to sperm DNA and apoptosis (12). It is now proved that vitamin E, as a potent antioxidant (13), protects the organism against oxidative stress via the inhibition of propagation of ROS reactions. In reproductive system, the antioxidant role of vitamin E has also been reported to reduce testicular oxidative stress (14). The antioxidant activity of vitamin E can improve diabetes-induced free radical damage in testicular tissue.

Therefore, this study aims at evaluating the effect of vitamin E on oxidative stress induced by diabetes in mice sperm.

## 2. Materials and Methods

### Animals and treatments 

In this experimental study, 30 Syrian adult male mice (30–40 gr) were divided equally into three groups - control, treated by vitamin E, and untreated group. For the induction of diabetes, 20 mice were intraperitoneally injected by a single dose (200 mg/kg) of Streptozotocin; 48 hours after the injection, blood glucose was measured by a glucometer (ACCU-CHEK - Germany) and the mice with blood glucose concentrations 200 to 350 mg/dl were accepted as being diabetic. In the treated group, vitamin E was intraperitoneally given to the mice for 35 days. At the end of this period, the animals were anesthetized and sacrificed by Dislocate. Testis of the animals were dissected and homogenized in phosphate buffer (10%, pH= 7.6). Then, the homogenized tissue was centrifuged at 10000 g for 15 min. The acquired supernatant was used for measuring the of Total Antioxidant Capacity (TAC) and malondialdehyde (MDA) as well as the evaluation of sperm parameters.

### Evaluation of sperm parameters

For counting sperms, Neubauer hemocytometer was used. Briefly, the dissected epididymis was transferred into Ham's F10 medium and used for sperm count by a microscope. To investigate the sperm motility, the sperm solution was placed on semen analysis chamber and evaluated by microscopic fields. The sperm morphology was evaluated by eosin-nigrosine-stained slides.

### MDA concentration

So as to determine MDA, 10 µl of hydroxy toluene was mixed with 100 µl sperm sample and 500 µ TAC. This mixture was vortexed for 10 minutes and then centrifuged in 3000 g. The supernatant was transferred to a microtube and mixed with 400 µl of thiobarbiturate acid and incubated for 1 h in bain-marie. After this period of time, it was placed in 4${}^{\circ }$C for 15 min and centrifuged at 4000 g for 10 min. The absorbance of supernatant was read at 532 and 573 nm. The difference between the two wavelengths was considered as the absorbance of sample. The MDA concentration was calculated based on acquired absorbance data.

### Total antioxidant capacity 

Total antioxidant capacity is considered as a balance between free radicals and antioxidants. To evaluate this parameter, radical scavenging activity is assessed through DPPH radical scavenging assay. 20 µl of plasma and 380 µl of phosphate buffer were poured into a micro tube. This solution was then mixed with 400 µl of methanolic solution of DPPH (0.15 mM in methanol). After 5 hr of incubation in room temperature, the absorbance of the mixture was measured at 520 nm. The control contained 400 µl of methanol and 400µl of DPPH. DPPH radical scavenging ability was calculated using the following equation:

white% reduction  of  DPPH white=(A control −−A sample )/A sample −−100.

### Determination of apoptosis

For the determination of apoptosis, the TUNEL method was used based on similar articles. In this method, the TdT enzyme is able to mark the 3-OHs that have been released into the free form by breaking the DNA in the course of apoptosis. Then, Fluorescein-containing places are identified by Anti-Fluorescein antibody attached to peroxidase. In this method, situ cell death detection kit and fluorescent microscope were used for the detection of sperms that were involved in apoptosis.

In this project, ethical principles in animal studies were observed and approved by the ethics committee of Shahid Sadoughi University of Medical Sciences with code number ir.ssu.medicin.rec.1395.36.

### Statistical analysis

To analyze MDA, TAC, chromatin density, and apoptosis, one-way ANOVA was applied, and to compare control and case groups, the statistical software of SPSS (Statistical Package for the Social Sciences, version 18.0, SPSS Inc, Chicago, Illinois, USA) and ANOVA tests were used. Also, the Tukey test was performed and the significance level of the test was considered to be less than 0.05.

## 3. Results

### Sperm count

Sperm count was done in the three groups and acquired data are summarized in Table I. Based on the data, highly significant decrease was observed in diabetic mice in comparison with control group (478×10${}^{5}$ and 171×10${}^{5}$ for control and untreated diabetic group, respectively). Having considered the number of sperms in the untreated and treated groups, it was found out that vitamin E leads to a remarkable increase in sperm number. There was a great difference between control and treated diabetic group (p= 0.026) (Table II).

### Sperm motility 

Compared to the control group, animals in diabetic group showed a remarkable increase in the percentage of immotile sperm (Table I). Mice treated with vitamin E showed a decrease in the percentage of immotile sperm in comparison with the untreated group. This increase was statistically significant (p= 0.026). There was also a great difference between treated and control group in terms of the percentage of immotile sperm (p= 0.024) (Table II). Also, we examined the sperm motility in the three groups and the data are summarized in Table I. The Bonferroni test demonstrated that there was a significant difference between control and untreated groups as well as treated and untreated groups in terms of slow and quick variations, but no significant difference was seen between the control and treated groups (Table II).

### Normal and abnormal sperm morphology

As shown in Table I, diabetic mice have low rate of normal sperm. This morphological anomalies was significant in comparison with the control group (p= 0.0001). The untreated group showed a significant increase (p< 0.0001) in abnormal sperm compared to control and treated groups. The quantity of normal sperm in treated group indicated that vitamin E could remarkably (p= 0.0001) reverse the sperm morphological anomalies as compared with the untreated group so that there was no significant difference between the control and treated groups (p= 0.091) (Table II).

### TAC 

According to the results in Table I, the TAC was strikingly decreased in diabetic mice. Vitamin E could dramatically improve TAC since its application did solve the problem to some extent, but the effect was not notable in comparison with untreated diabetic mice. The control group and treated diabetic group (p= 1) were almost similar in TAC.

### MDA concentration

The concentration of MDA in all three groups was similar (Table I), that is, there is not much difference between these groups in terms of MDA concentration (p> 0.05) (Table II). So, diabetes and its related factors had no effect on the MDA concentration.

### Apoptosis assay

The effect of vitamin E on cell apoptosis was evaluated by TUNEL method, and its acquired results were summarized in Table I. According to the table, there was a significant difference between the groups. For a more accurate analysis, this factor was compared between each two groups (as shown in Table II). There was a remarkable difference between the control and untreated groups. But, this variable was not considerable between the control and treated groups. The treatment of the mice with vitamin E improved this variable and reduced the cell apoptosis.

**Table 1:** Examined variables (sperm number, sperm morphology, TAC, MDA concentration, sperm motility, apoptosis) among the three groups.


**Variable**	**Group**		
	**Control**	**Untreated Group**	**Treated Group**
Number of sperm	478 × 10${}^{5}$	171 × 10${}^{5}$	322 × 10${}^{5}$
Normal Morphology	78 ± 5.87	49.6 ± 31.93	70.2 ± 7.46
Abnormal Morphology	22 ± 5.66	49.4 ± 9.77	29.8 ± 7.47
TAC*	24.74 ± 10.34	12.03 ± 11.98	8.48 ± 9.89
MDA*	2.47 ± 0.27	2.55 ± 0.28	2.66 ± 0.11
Slow	17.5 ± 3.54	10.7 ± 7.48	18.7 ± 5.98
Quick	25 ± 12.92	1 ± 4.65	12.5 ± 5.67
Immutile	58 ± 3.53	86.4 ± 7.48	70.3 ± 5.95
TUNEL* medium (apoptosis indicator)	4.5	27	10
Data presented as Note: *TAC: Total Antioxidant Capacity; *MDA: malondialdehyde; *TUNEL: Terminal deoxynucleotidyl transferase dUTP nick end labeling; ANOVA test was used for analysis.			

**Table 2:** Comparison of statistical analysis of examined variables (sperm number, sperm morphology, TAC, MDA concentration, sperm motility, apoptosis) among the three groups.


**Variable**	P**-value Between Groups**		
	**Control & Untreated Groups**	**Treated & Untreated Groups**	**Control & Treated Groups**
Number of sperm	0.0001	0.026	0.048
Normal Morphology	0.0001	0.091	0.0001
Abnormal Morphology	0.0001	0.102	0.0001
TAC*	0.041	0.007	1
MDA*	1	0.08	0.11
Slow	0.047	1	0.016
Quick	0.0001	0.005	0.069
Immutile	0.0001	0.024	0.026
TUNEL* (apoptosis indicator)	0.0001	0.066	0.025
Note: *TAC: Total Antioxidant Capacity; *MDA: malondialdehyde; *TUNEL: Terminal deoxynucleotidyl transferase dUTP nick end labeling; ANOVA test was used for analysis.			

## 4. Discussion

In this study, the effect of vitamin E on sperm parameters in adult diabetic mice was evaluated. The present study demonstrated that testicular characteristics including number of normal sperm, mobility, morphology, and apoptotic cells change in diabetic mice. These changes were associated with enhanced oxidative stress and apoptosis in the testes of diabetic mice (15). It was proved that diabetes causes testicular dysfunction and also has some effects on testicular parameters and reproductive system such as increase in semififerous tubule thickness, reduction in germ cells population, vacule formation in Sertoli cells, the decrease in volume of testis and semen, decrease in Leydig cells and Testosterone (16). The majority of these effects are related to oxidative stress in a diabetic person (17). The increase of blood sugar may result in the increase of free radical formation and reduce endogenous antioxidant capacity that leads to enhanced oxidative stress in testicular tissue. In another study, Hulya *et al*. showed that DM may cause testicular damage due to ROS elevation. They also demonstrated that Vit E can improve testicular damages. They concluded that, the high levels of ROS in DM inhibits steroidogenesis via different mechanisms, without causing any histopathologic change (18).

The overproduction of ROS causes mitochondrial damage and lipid peroxidation in testicular cells and results in dysfunction of testicular spermatogenesis (19). The acquired data from this study also indicated the effect of diabetes on sperm parameters. Koksal also showed that lipid peroxidation cause sever pathological alterations in testis. Their results confirm that high levels of may have a critical role in testicular degeneration and infertility (20). Mustafa Sönmez *et al*. revealed that chronic treatment of Hcy has deleterious effect on the epididymal sperm parameters in male Mice. They also showed that the administration of Melatonin or Vitamin E can protect spermatozoa and Leydig cells from adverse effects of Hcy in Mice (21).

Mangoli and colleagues. presented that the sperm parameters in diabetic mice are significantly lower than in normal mice. The main reason for the reduction of these parameters is the oxidative stress induced by diabetes, and the toxic effect of diabetes on the sperm number and morphology can be a result of diabetes-induced stress oxidative (22).The most important effect of diabetes is the production of free radicals and their related stress oxidative (23). Vitamin E can reverse the hazardous effect of diabetes on sperm number, morphology, and other related parameters. The results of this study showed that in the treated diabetic group, vitamin E remarkably eliminated diabetes-mediated reduction of sperm number. Vitamin E application reduced the side effects of diabetes and enhanced the total sperm number to normal range, to some extent (24). The results of this study is in line with those of Parizadian Kavan and colleagues. and Azawi and colleagues. (25, 26). Parizadian Kavan showed that the use of vitamins E and C in tris extender and the use of vitamin E in milk extender is recommended for the storage of Atabay ram semen in liquid conditions with high quality of their parameters (25). Azawi also demonstrated that the addition of the antioxidants like Vit C and Vit E to the preservation media can improve sperm quality in Awassi ram (26). in the way that vitamin E reduces the destructive effects of oxidative stress on sperm, and these antioxidant properties improve sperm parameters such as sperm morphology (27–29). Also, its effect on free radicals leads to sperm protection from apoptosis induction. In this study, the reduction of apoptosis level confirms this matter. MDA concentration was assayed as a biomarker of lipid peroxidation. MDA concentration in untreated diabetic mice was not significantly different from that of the control and treated diabetic mice; so, in spite of the sperm number, the MDA concentration remained unchanged in the three groups. This result suggests that the lipid peroxidation occurs in small amounts in diabetic mice. Numerous studies have proved that vitamin E has antioxidative properties as it improves TAC, suppresses destructive oxygen free radicals, and prevents oxidative stress damage (30).

In this article, we also demonstrate that the use of vitamin E significantly increases TAC in the treated diabetic mice compared to the untreated diabetic mice. In the treated diabetic mice, TAC was still lower than the normal groups. So, vitamin E improves the sperm parameters inducing oxidative stress and their harmful effects on testis.

## 5. Conclusion

As a conclusion, the study indicates that vitamin E has potent effects as antioxidant agent in the reduction of adverse side effects of diabetes-induced stress oxidative on sperm parameters. The use of vitamin E may be useful for the elimination of complications resulting from oxidative stress in diabetes.

##  Conflict of Interest

The authors declare that they have no conflict of interests.

## References

[1] Dinulovic D., Radonjic G. (1990). Diabetes mellitus/male infertility. *Systems Biology in Reproductive Medicine*.

[2] Matough FA., Budin SB., Hamid ZA., Alwahaibi N., Mohamed J. (2012). The role of oxidative stress and antioxidants in diabetic complications. *Sultan Qaboos Univ Med J*.

[3] Alves MG., Martins AD., Rato L., Moreira PI., Socorro S., Oliveira PF. (1832). Molecular mechanisms beyond glucose transport in diabetes-related male infertility. *Biochim Biophys Acta2013*.

[4] Romano A. D., Serviddio G., De Matthaeis A., Bellanti F., Vendemiale G. (2010). Oxidative stress and aging. *Journal of Nephrology*.

[5] Elmore S. (2007). Apoptosis: a review of programmed cell death. *Toxicologic Pathology*.

[6] Oberley L. W. (1988). Free radicals and diabetes. *Free Radical Biology & Medicine*.

[7] Lobo V., Patil A., Phatak A., Chandra N. (2010). Free radicals, antioxidants and functional foods: impact on human health. *Pharmacognosy Reviews*.

[8] Lü J.-M., Lin P. H., Yao Q., Chen C. (2010). Chemical and molecular mechanisms of antioxidants: experimental approaches and model systems. *Journal of Cellular and Molecular Medicine*.

[9] Bajaj S., Khan A. (2012). Antioxidants and diabetes. *Indian J Endocrinol Metab*.

[10] Agarwal A., Allamaneni S. S. R. (2011). Free radicals and male reproduction. *Journal of the Indian Medical Association*.

[11] Henkel R. R. (2011). Leukocytes and oxidative stress: dilemma for sperm function and male fertility. *Asian Journal of Andrology*.

[12] RODRIGUES B., CAM M., MCNEILL J. (1995). Myocardial substrate metabolism: Implications for diabetic Cardiomyopathy. *Journal of Molecular and Cellular Cardiology*.

[13] Sanders R. A., Rauscher F. M., Watkins J. B. (2001). Effects of quercetin on antioxidant defense in streptozotocin-induced diabetic rats. *Journal of Biochemical and Molecular Toxicology*.

[14] Pan X.-R., Li G.-W., Hu Y.-H., Wang J.-X., Yang W.-Y., An Z.-X., Hu Z.-X., Lin J., Xiao J.-Z., Cao H.-B., Liu P.-A., Jiang X.-G., Jiang Y.-Y., Wang J.-P., Zheng H., Zhang H., Bennett P. H., Howard B. V. (1997). Effects of diet and exercise in preventing NIDDM in people with impaired glucose tolerance: the Da Qing IGT and diabetes study. *Diabetes Care*.

[15] Johansen JS., Harris AK., Rychly DJ., Ergul A. (2005). Oxidative stress and the use of antioxidants in diabetes: linking basic science to clinical practice. Cardiovasc Diabetol. *Ergul A. Oxidative stress and the use of antioxidants in diabetes: linking basic science to clinical practice. Cardiovasc Diabetol*.

[16] Esposito K., Maiorino M. I., Bellastella G. Diabetes and sexual dysfunction: current perspectives. *Diabetes, Metabolic Syndrome and Obesity: Targets and Therapy*.

[17] Tang X., Zhang Q., Dai D., Ying H., Wang Q., Dai Y. (2008). Effects of strontium fructose 1,6-diphosphate on expression of apoptosis-related genes and oxidative stress in testes of diabetic rats. *International Journal of Urology*.

[18] Aybek H., Aybek Z., Rota S., Şen N., Akbulut M. (2008). The effects of diabetes mellitus, age, and vitamin E on testicular oxidative stress. *Fertility and Sterility*.

[19] Spirlandeli A., Deminice R., Jordao A. (2014). Plasma Malondialdehyde as Biomarker of Lipid Peroxidation: Effects of Acute Exercise. *International Journal of Sports Medicine*.

[20] Koksal I. T., Usta M., Orhan I., Abbasoglu S., Kadioglu A. (2003). Potential role of reactive oxygen species on testicular pathology associated with infertility. *Asian Journal of Andrology*.

[21] Sönmez M., Yüce A., Türk G. (2007). The protective effects of melatonin and Vitamin E on antioxidant enzyme activities and epididymal sperm characteristics of homocysteine treated male rats. *Reproductive Toxicology*.

[22] Mangoli E., Talebi A. R., Anvari M., Pourentezari M. (2013). Effects of experimentally-induced diabetes on sperm parameters and chromatin quality in mice. *Iranian Journal of Reproductive Medicine*.

[23] Ding G., Liu Y., Liu M., Pan J., Guo M., Sheng J., Huang H. (2015). The effects of diabetes on male fertility and epigenetic regulation during spermatogenesis. *Asian Journal of Andrology*.

[24] Asmat U., Abad K., Ismail K. (2015). Diabetes mellitus and oxidative stress—a concise review. *Saudi Pharmaceutical Journal*.

[25] Parizadian Kavan B., Jafari Ahangari Y., Zerehdaran S. (2008). [The effects of various levels of vitamins E and C in milk and tris extenders on characteristics of Atabay ram semen in liquid condition.] J Agric Sci Natur Resour. *Zerehdaran S. [The effects of various levels of vitamins E and C in milk and tris extenders on characteristics of Atabay ram semen in liquid condition.] J Agric Sci Natur Resour*.

[26] Azawi OI., Hussein EK. (2013). Effect of vitamins C or E supplementation to Tris diluent on the semen quality of Awassi rams preserved at 5║C. Vet Res Forum. *Hussein EK. Effect of vitamins C or E supplementation to Tris diluent on the semen quality of Awassi rams preserved at 5ºC. Vet Res Forum*.

[27] Mortazavi M., Salehi I., Alizadeh Z., Vahabian M., Roushandeh A. M. (2014). Protective effects of antioxidants on sperm parameters and seminiferous tubules epithelium in high fat-fed rats. *Journal of Reproduction and Infertility*.

[28] Zarei L., Sadrkhanlou R., Shahrooz R., Malekinejad H., Eilkhanizadeh B., Ahmadi A. (2014). Protective effects of vitamin E and cornus mas fruit extract on methotrexate-induced cytotoxicity in sperms of adult mice. Vet Res Forum. *Ahmadi A. Protective effects of vitamin E and cornus mas fruit extract on methotrexate-induced cytotoxicity in sperms of adult mice. Vet Res Forum*.

[29] Bansal Amrit Kaur, Bilaspuri G. S. (2011). Impacts of Oxidative Stress and Antioxidants on Semen Functions. *Veterinary Medicine international*.

[30] Li S., Tan H. Y., Wang N., Zhang Z. J., Lao L., Wong C.-W., Feng Y. (2015). The role of oxidative stress and antioxidants in liver diseases. *International Journal of Molecular Sciences*.

